# Catalytic Hydrolysis Mechanism of Cocaine by Human Carboxylesterase 1: An Orthoester Intermediate Slows Down the Reaction

**DOI:** 10.3390/molecules24224057

**Published:** 2019-11-09

**Authors:** Maocai Yan, Zhen Zhang, Zhaoming Liu, Chunyan Zhang, Jingchang Zhang, Shuai Fan, Zhaoyong Yang

**Affiliations:** 1 School of Pharmacy, Jining Medical University, Rizhao 276800, China; 2 Beijing Key Laboratory of Antimicrobial Agents, Institute of Medicinal Biotechnology, Chinese Academy of Medical Sciences and Peking Union Medical College, Beijing 100050, China; 3 Institute of Medicinal Biotechnology, Chinese Academy of Medical Sciences and Peking Union Medical College, Beijing 100050, China

**Keywords:** human carboxylesterase 1, catalytic mechanism, QM-cluster, novel intermediate, drug metabolism

## Abstract

Human carboxylesterase 1 (hCES1) is a major carboxylesterase in the human body and plays important roles in the metabolism of a wide variety of substances, including lipids and drugs, and therefore is attracting more and more attention from areas including lipid metabolism, pharmacokinetics, drug–drug interactions, and prodrug activation. In this work, we studied the catalytic hydrolysis mechanism of hCES1 by the quantum mechanics computation method, using cocaine as a model substrate. Our results support the four-step theory of the esterase catalytic hydrolysis mechanism, in which both the acylation stage and the deacylation stage include two transition states and a tetrahedral intermediate. The roles and cooperation of the catalytic triad, S221, H468, and E354, were also analyzed in this study. Moreover, orthoester intermediates were found in hCES1-catalyzed cocaine hydrolysis reaction, which significantly elevate the free energy barrier and slow down the reaction. Based on this finding, we propose that hCES1 substrates with β-aminocarboxylester structure might form orthoester intermediates in hCES1-catalyzed hydrolysis, and therefore prolong their in vivo half-life. Thus, this study helps to clarify the catalytic mechanism of hCES1 and elucidates important details of its catalytic process, and furthermore, provides important insights into the metabolism of hCES1 substrates and drug designing.

## 1. Introduction

Carboxylesterases (CES) are a large class of enzymes responsible for hydrolysis of esters and amides, and play very important roles in the metabolism of various drugs and biosubstances [1,2,3,4,5,6,7]. According to their sequence homology, CES can be generally divided into several classes, and the majority of known CES belong to CES1 and CES2 classes [3,8]. In the human body, human carboxylesterase 1 (hCES1) and carboxylesterase 2 (hCES2) have distinct tissue distributions and very different substrate specificities, and are known to be responsible for the metabolism of different substances or drugs [3,6,8]. hCES2 mainly exists in the gastrointestinal tract and a lower level in liver, while hCES1 distributes mainly in liver, and less in intestine, kidney, and other tissues. It is known that hCES1 and hCES2 have significantly different substrate specificities: hCES1 mainly prefers ester substrates with a large acyl group and a small alcohol group, whereas hCES2 favors substrates with a small acyl group and a large alcohol group [6,8]. As an important esterase in the human body, hCES1 participates in the metabolism of many endogenous lipids, including cholesterol and fatty acid esters, as well as a great many commonly used drugs (including cocaine, clopidogrel, oseltamivir, methylphenidate, meperidine, enalapril, etc.) [3,4,5,6]. Because of its important roles in metabolism of lipids and drugs, hCES1 is now attracting more and more attention from researchers in multiple disciplines, including lipid metabolism, prodrug design, drug metabolism, pharmacokinetics, drug–drug interactions, etc. [5,6,7,8,9].

Since hCES1 is a crucial enzyme for the metabolism of many important lipids and drugs, it would be very valuable to clearly understand its catalytic mechanism of substrate hydrolysis reaction, to increase our understanding of properties of carboxylesterases, such as substrate-specificity and reaction rates, as well as to facilitate the development of drugs or prodrugs which need to be metabolized or activated by hCES1. It has been generally proposed that the reaction process of carboxylic ester hydrolysis catalyzed by esterases can be divided into two stages: (I) Formation of acyl–enzyme intermediate and release of alcohol (“the acylation stage”), and (II) hydrolysis of acyl–enzyme intermediate to give free enzyme and carboxylic acid (“the deacylation stage”) [2,10,11]. In recent years, theoretical computation studies, mainly using quantum mechanics (QM) or a hybrid of quantum mechanics/molecular mechanics (QM/MM) methods, have been performed to elucidate the detailed catalysis mechanism of esterases, including acetylcholinesterase (AChE) [12], butyrylcholinesterase (BChE) [13], cocaine esterase (CocE) [14], and triacylglycerol lipase [15,16]; these studies revealed that each of the two stages (the acylation stage and the deacylation stage) consists of two steps: The nucleophilic addition (of the catalytic residue or water) and the elimination (of the alcohol or free catalytic residue), separated by a stable tetrahedral intermediate.

As far as carboxylesterase 1 (CES1) is concerned, in recent years, several crystalline structures of hCES1 (and CES1 from other species) complexed with different substrates have been solved [17,18,19], and some details of the catalytic hydrolysis mechanism have been explored based on the crystal structures and theoretical computations using density functional theory (DFT) or the QM/MM method [19,20,21]. For instance, Aranda et al. [20] performed a DFT study on the hydrolysis mechanism of ester substrates catalyzed by AeCXE1 (a CES1 from *Actinidia eriantha*) and revealed a four-step mechanism, in which both the formation and the hydrolysis of the acy–lenzyme intermediate consist of two elementary reactions. In 2017, Wang et al. [21] simulated the acylation stage of hCES1-catalyzed hydrolysis of methylphenidate using QM/MM, and found the formation of acyl–hCES1 complex to be a two-step process, including two transition states (TS) separated by a tetrahedral intermediate (IM). These results agree with the previous mechanism studies of other esterases.

However, a few recent studies have challenged the four-step catalytic hydrolysis mechanism of esterases. In 2016, a QM/MM study on the hydrolysis mechanism of ghrelin catalyzed by butyrylcholinesterase [22] found the acylation stage to be a single-step process with no tetrahedral intermediate. Again, in 2018, a two-step mechanism of hCES1-catalyzed hydrolysis of cocaine was presented [23], in which both the acylation stage and the deacylation stage are single-step processes (with no tetrahedral intermediates). These findings urged us to further explore the catalytic mechanism of esterases, in order to clarify the catalytic mechanisms and elucidate more details in the enzymatic reactions, which would be helpful in the study of drugs or bioactive substances metabolized by hCES1 or other esterases. In this work, we present a DFT computation study on the hCES1-catalyzed hydrolysis mechanism, using cocaine as the model drug, and compare our results with previous QM/MM results to get more insights into the catalytic mechanism of hCES1.

## 2. Results and Discussions

### 2.1. Gibbs Free Energy Profile of the Whole Reaction Pathway

**Figure 1:**
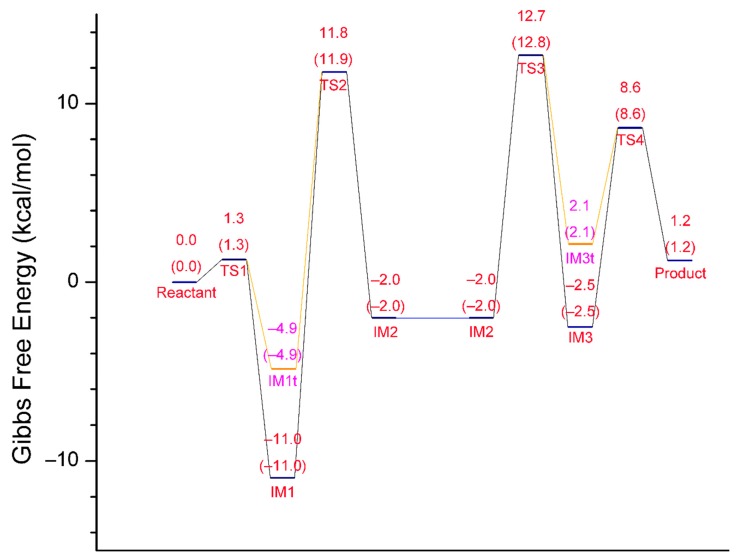
Free energy profile of the whole reaction process of hCES1-catalyzed hydrolysis of cocaine. The relative Gibbs free energies at 298 K and 310 K of each substance are given in kcal/mol (the numbers in parentheses are the free energies at 310 K). IM1 and IM3 are orthoester intermediates, whereas IM1t and IM3t are tetrahedral intermediates before the formation of orthoesters. The left half (Reactant → IM2) is the process of acylation of hCES1, wherein IM2 is the complex of acyl–hCES1 with a methanol molecule; the right half (IM2 → Product) is the process of hydrolysis of acyl–hCES1 intermediate, wherein IM2 indicates the complex of acyl–hCES1 with a water molecule.

**Figure 2:**
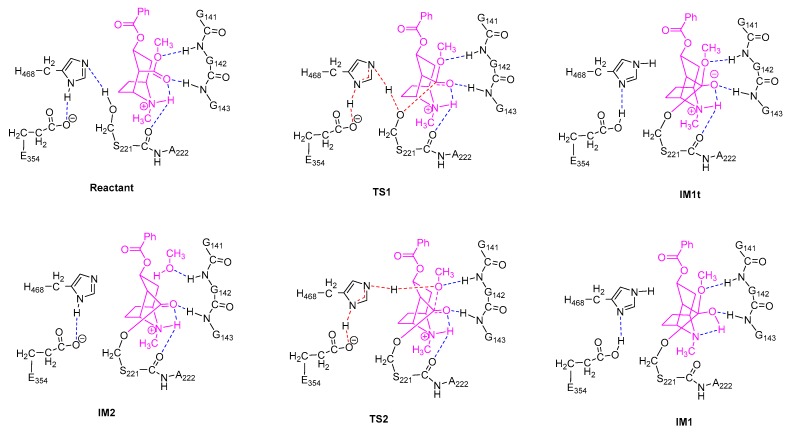
Transition states and intermediates in the acylation stage of hCES1. The ligands are shown in magenta. Non-polar residues which are far from the reaction center are not displayed. Blue dashed lines indicate common hydrogen bonds, and red/magenta dashed lines are bonds to be formed or broken.

**Figure 3:**
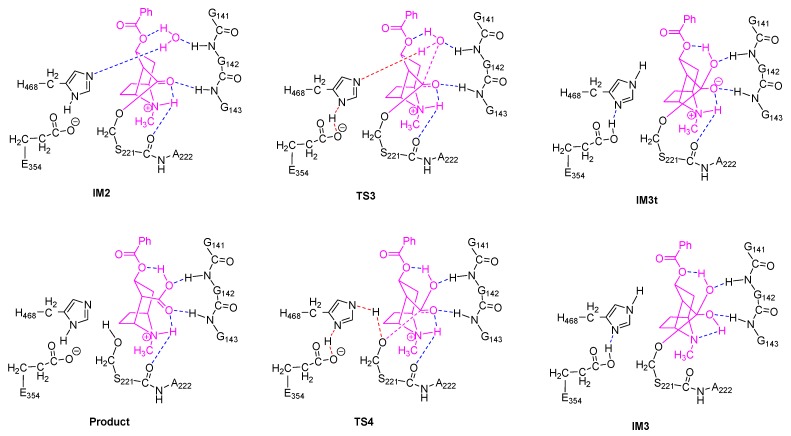
Transition states and intermediates in the deacylation stage of hCES1.

For each of the two stages of the catalytic hydrolysis, i.e., acylation of hCES1 and deacylation of hCES1, the transition states were found and the intermediates, reactant, and product were obtained through intrinsic reaction coordinate (IRC) calculation. None of the intermediates, reactant, or product had imaginary frequencies in the vibrational analysis, while each of the transition states had only one imaginary frequency; this indicates that the freezing of C_α_ atoms during the computations did not introduce inappropriate restraints which would significantly interfere with the catalysis process. The Gibbs free energy of each of the stationary point structures are illustrated in the free energy profile in Figure 1, which clearly reveal that each of the two stages was a two-step process. During the acylation stage of hCES1, the catalytic residue Ser221 attacked the carbonyl carbon of the substrate cocaine to form an orthoester intermediate IM1, which was then cleaved to give acyl–hCES1 and a methanol molecule (IM2) (Figure 2). During the deacylation stage, a water molecule attacked the acyl–hCES1 complex to form another orthoester intermediate, IM3, and then the ester bond between acyl and Ser221 was broken to release the free enzyme and hydrolysis product (Figure 3). (Please see Electronic Supplementary Information for the detailed structures.)

### 2.2. Acylation Stage of hCES1-Catalyzed Cocaine Hydrolysis

In the initial structure of hCES1-cocaine complex (Reactant, Figure 2), cocaine bound to the catalytic site and formed three hydrogen bonds to hCES1, i.e., cocaine:NH…S221:O, cocaine:C=O…G143:NH, and cocaine:CH_3_O…G142:NH. These three hydrogen bonds retained in all transition states and intermediates in the acylation stage, except IM1, the orthoester intermediate. Lipophilic interactions also formed between cocaine and the adjacent non-polar residues (L97, L255, L363, and F426), which are not displayed in Figure 2 and Figure 3 for clarity. During the first elementary reaction, nucleophilic addition of S221:O^γ^ to cocaine carbonyl group, S221:H^γ^ gradually moved from O^γ^ to H468:N^ε^, while S221:O^γ^ approached cocaine carbonyl carbon to form a “triangle pyramid” transition state (TS1). The Gibbs free energy barrier of this step was quite low (1.3 kcal/mol only) (Figure 1), indicating that TS1 was easily formed and possibly closed to the structure of Reactant. The distance between S221:O^γ^ and carbonyl carbon was 1.995 Å, much longer than common C–O single bond length; the C=O and C–OMe bond lengths were 1.232 Å and 1.364 Å, respectively, similar to those in Reactant (1.211 Å and 1.339 Å, respectively). The carbonyl oxygen formed one hydrogen bond with G143:NH and another hydrogen bond with cocaine:NH to form an intramolecular six-membered ring, and therefore became more stable. After that, S221:O^γ^ continued to approach the carbonyl carbon to form a tetrahedral intermediate (IM1t). During the conversion of Reactant → TS1 → IM1t, S221:H^γ^ was transferred from S221:O^γ^ to H468:N^ε^, and H468:H^δ^ was transferred from H468:N^δ^ to E354 carboxylate anion to form a carboxylic acid, accompanying the electron rearrangement in the histidine ring of H468. Through such a proton transfer and nucleophilic addition, the negative formal charge was transferred from E354 in Reactant to cocaine carbonyl oxygen in IM1t. This S221–H468–E354 proton transferring chain found in this study agrees very well with the previous theories [4,24,25].

**Figure 4:**
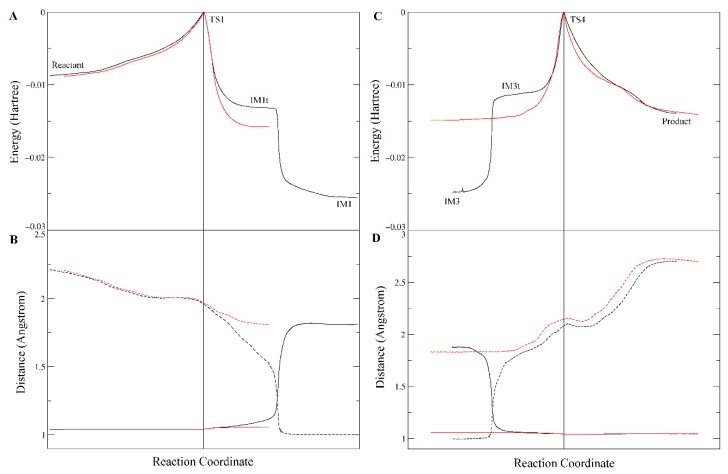
Intrinsic reaction coordinate of TS1 (**A**,**B**) and TS4 (**C**,**D**). (**A**,**C**) Changes of single point energies along the reaction coordinate; (**B**,**D**) Distances between cocaine N and NH (solid lines) and between carbonyl O and NH (dash lines). The red lines are the computation results with no polarization function for hydrogens, while the black ones are the normal results.

However, interestingly, IM1t was not the final product of this step. As illustrated in the IRC of TS1 (Figure 4, the black lines), after formation of IM1t, cocaine:NH gradually migrated from the nitrogen toward the carbonyl oxygen, and finally formed an orthoester tetrahedral intermediate IM1 with no formal charges on nitrogen or oxygen atoms. The orthoester intermediate IM1 had the lowest Gibbs free energy (−11.0 kcal/mol) among all the stationary point structures, significantly lower than IM1t (−4.9 kcal/mol). The higher stability of IM1 over IM1t can be attributed to the fact that the carbonyl oxygen atom formed two formal covalent bonds and had no formal negative charges, and the conversion from IM1t to IM1 was actually an acid-base neutralization in which the “acid” R_3_NH^+^ donated a proton to the “base” oxygen anion.

It is also worth mentioning that polarization functions of hydrogen atoms were necessary for the calculation of this proton transfer. Although it is common sense that polarization functions of hydrogen are needed in theoretical studies of chemical reactions involving hydrogen transfer, in many DFT and QM/MM studies of large systems like protein or enzymatic reactions, polarization functions of hydrogen were usually omitted to reduce the computation workload. To evaluate the effects of this omission on the DFT computation results of our system, we removed the polarization functions of hydrogen atoms involved in the proton transfer and hydrogen bonding (i.e., by changing their basis set from 6–31G** to 6–31G*). As shown in Figure 4 (the red lines), the proton transfer process of S221 → H468 → E354 was not significantly affected; however, the IRC terminated at the tetrahedral intermediate IM1t and would not proceed to the orthoester IM1. This result might indicate that the polarization functions of hydrogen are important in theoretical studies of such kind of enzymatic reactions and could be useful in exploring more details of the reaction mechanism.

In the next step, H468:N^ε^ coordinated cocaine:OMe through its H^ε^ (which was just S221:H^γ^, received from S221) to form the transition state TS2, and some conformation transitions occurred in cocaine and sidechains of residues including H468 and S221 during this process. TS2 had a relative Gibbs free energy of 11.8 kcal/mol, and the free energy barrier of this reaction was 22.7 kcal/mol (22.8 kcal/mol read directly from Figure 1, due to rounding errors). In TS2, cocaine:OMe was rather far from the carbonyl carbon (2.060 Å), and the carboxylester group of acyl–hCES1 was closed to a planar conformation; this indicates that the tetrahedral intermediate IM1 needed to overcome much resistance to form TS2 to facilitate the leaving of the methoxy group. Starting from TS2, H468:N^ε^ gradually transferred its H atom to cocaine:OMe, which gradually departed from the carbonyl carbon and finally cleaved from the cocaine main body as a methanol molecule (as displayed in IM2). And during this process, E354 transferred the proton back to H468:N^δ^.

In summary, the acylation stage consisted of two steps, and the second step determined the rate of the whole acylation process with a free energy barrier of 22.7 kcal/mol. During this stage, S221:O^γ^ transferred its proton to H468:N^ε^ which then passed the proton to cocaine:OMe; at the same time, S221:O^γ^ approached the carbonyl carbon to form a tetrahedral orthoester intermediate, then the methoxy group (together with the received proton) left the carbonyl carbon to release a methanol molecule. Synchronous with the receiving and donating proton of H468:N^ε^, H468:H^δ^ was transferred between H468:N^δ^ and E354:carboxylate in concert with bond rearrangement of the imidazole ring. These details agree well with the previous hypotheses [4,24,25].

### 2.3. Deacylation Stage of hCES1-Catalyzed Cocaine Hydrolysis

The deacylation process, as illustrated in Figure 3, was basically a reverse process of the acylation stage. Starting from the acyl–hCES1 complex IM2, in which a water molecule replaced the methanol molecule in Figure 2, the water molecule approached the carbonyl carbon to form a triangle pyramid transition state, TS3, and simultaneously, a hydrogen atom of the water molecule moved away from the water oxygen towards H468:N^ε^. In TS3, the distance between water oxygen and carbonyl carbon was 1.988 Å. After crossing TS3, the water oxygen continued to approach the carbonyl carbon to form a tetrahedral intermediate, which then converted to an orthoester intermediate, IM3. At the same time, the water molecule transferred a proton to H468:N^ε^, while H468:N^δ^ transferred its proton to E354 carboxylate anion to form a carboxylic acid. The Gibbs free energy barrier of this step was 14.7 kcal/mol.

In the fourth step, S221:O^γ^ moved away from the carbonyl carbon to form the triangle pyramid transition state TS4, in which the distance between S221:O^γ^ and the carbonyl carbon was 2.001 Å. Then, S221:O^γ^ continued leaving the carbonyl carbon and received a proton from H468:N^ε^ (which was originally from the water molecule) to restore the free catalytic S221 residue. Synchronously with the H468 → S221 proton transfer, E354 carboxylic acid transferred a proton to H468 and converted to a carboxylate anion. The free energy barrier of this step was 11.1 kcal/mol.

Therefore, briefly, during the deacylation stage, a water molecule added to the carbonyl carbon to form a tetrahedral intermediate, then S221:O^γ^ left the carbonyl carbon to release the free S221 residue and cocaine carboxylic acid; in this process, the water molecule transferred one of its protons to H468, which then passed it to S221:O^γ^, and another proton transferred back and forth between E354 and H468 in cooperation with the water → H468 → S221 proton transfer. These details also agree well with the previous hypotheses [4,24,25]. Step 3 determined the rate of the deacylation stage, and the free energy barrier was 14.7 kcal/mol.

In summary, the hCES1-catalyzed hydrolysis of cocaine consisted of four steps: Steps 1 and 2 were acylation of hCES1 and Steps 3 and 4 were deacylation of hCES1. Step 2 determined the overall rate of the whole enzymatic hydrolysis reaction, and the activation energy (*E*_a_) of the whole reaction was 22.7 kcal/mol. This value agrees well with the experimental results (*k*_cat_ = 0.058 min^−1^, *E*_a_ = 21.5 kcal/mol, 298 K) [23].

### 2.4. Differences Between Our Computational Results and Previous Results

Both the previous results [23] and ours gave good predicted *E*_a_ values of the hCES1-catalyzed hydrolysis of cocaine (20.1 kcal/mol and 22.7 kcal/mol, respectively), which were close to the experimental results (21.5 kcal/mol). However, many important differences were found between these studies.

**Figure 5:**
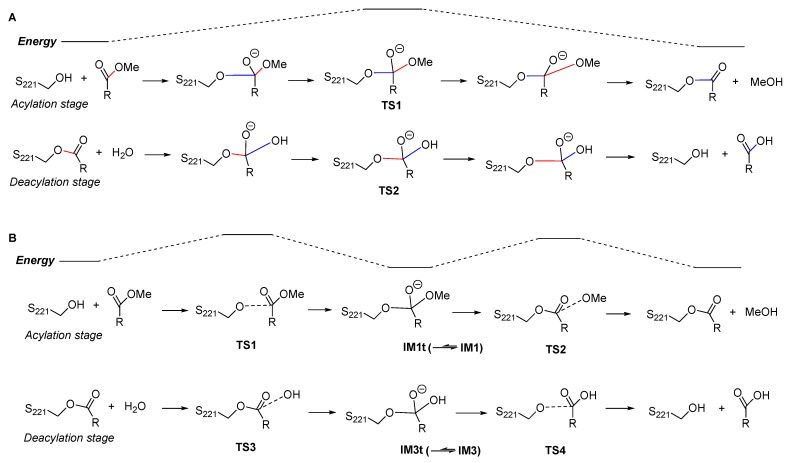
A brief comparison of previously reported cocaine hydrolysis mechanism (**A**) and our results (**B**). The blue bonds are newly formed bonds whose length is continuously decreasing, and the red bonds are bonds to be broken whose length is continuously increasing. For simplicity, the proton transfer and the involved catalytic residues (H468 and E354) are not shown here.

Firstly, the previous QM/MM studies suggested a two-step reaction mechanism in which both the acylation stage and the deacylation stage were a single-step process with no tetrahedral intermediates (Figure 5A). For the acylation process, it was actually a typical S_N_2 reaction in which the approaching of S221:O^γ^ to the carbonyl carbon was concerted with the leaving of methoxy group; when S221:O^γ^ reached an “equilibrium” with the methoxy group, the system reached a “watershed” at the energy maximum of the reaction coordinate, i.e., the tetrahedral transition state TS1. The deacylation process was similar to the acylation. Such an S_N_2 mechanism commonly occurred in alkyl substitutions, but was rarely found in interconversion of carboxylic acid derivatives, including transesterification and ester hydrolysis [26]. In contrast, our studies revealed a canonical four-step mechanism, in which both the acylation and the deacylation stages consisted of two steps, the nucleophilic addition and the elimination, with two triangular pyramid transition states separated by a tetrahedral intermediate (Figure 5B).

Generally, the S_N_2 mechanism was not favorable in the acyl substitutions, like transesterification and ester hydrolysis, because the leaving group alkoxide anion (herein, MeO^−^ or S221−O^−^) was a strong base, whereas the addition–elimination mechanism would facilitate the leaving of alkoxide anion by lowing the activation energy of the elimination step [26]. However, we could assume that an S_N_2 mechanism might be possible in the enzymatic ester hydrolysis reactions, since the catalytic residues (herein, H468, etc.) may coordinate the alkoxide group and facilitate its leaving process. To verify this hypothesis, we tried to search for the transition states of the S_N_2 reaction process, using the model and computation method as described here, but failed. The obtained “transition states” were very similar to the tetrahedral intermediates IM1t or IM3t, and their unique imaginary frequencies and “intrinsic reaction coordinates” did not lead to the desired reactants and products, but led to the orthoester intermediates (IM1 and IM3). These results indicate that such an S_N_2 mechanism was not possible using our computation method, and the assumed tetrahedral “transition states” were actually tetrahedral intermediates located at energy minima (if they would not further convert to orthoesters).

In our opinion, such different outcomes probably arose from the different models and computation methods used in these studies. Both the QM cluster approach and the QM/MM approach are widely used in the study of enzymatic reactions [27,28,29,30,31]. QM/MM simulations are usually considered to have advantages in modeling the long-range effects of the environments and simulating the high number of degrees of freedom of the system, and therefore could give better estimates of activation free energies (*E*_a_) [31]. Compared to the QM/MM method, the QM cluster method usually neglects ensemble averaging, which could be very important in enzyme kinetics [32]. However, due to the extremely high computation workload in QM/MM molecular dynamics simulations of enzymatic reactions, low computation levels (such as semi-empirical methods, e.g., SCC-DFTB and PM6) usually have to be applied to the QM region [30]. We noticed that the major parts of computation in the two works supporting the one-step acylation/deacylation mechanism [22,23] were performed with SCC-DFTB in combination of molecular force field CHARMM27. Actually, in a work published in 2017 [21], the hCES1-catalyzed hydrolysis mechanism of *d*-threo-methylphenidate was studied using SCC-DFTB and the results were compared with those of DFT (B3LYP/6–31G*). The DFT method clearly showed a two-step acylation process, with two transition states separated by a tetrahedral intermediate; however, the SCC-DFTB method overestimated the free energy of the intermediate, and therefore the intermediate had the highest free energy of the whole reaction pathway and thus became “the only transition state” of the whole acylation process, which seemed like a “one-step” acylation process. Later in the same work [21], the SCC-DFTB method was optimized by modifying the Mulliken charge and then the two-step acylation mechanism was reproduced using the modified SCC-DFTB (termed “SCC-DFTBMR”). In the computation study of hCES1-catalyzed hydrolysis of cocaine [23] and butyrylcholinesterase-catalyzed hydrolysis of ghrelin [22], the SCC-DFTB method was used without modifications. So, it might be possible that the free energy of tetrahedral intermediates of cocaine and ghrelin were also overestimated, and hence, they seemed like “tetrahedral transition states”.

**Figure 6:**
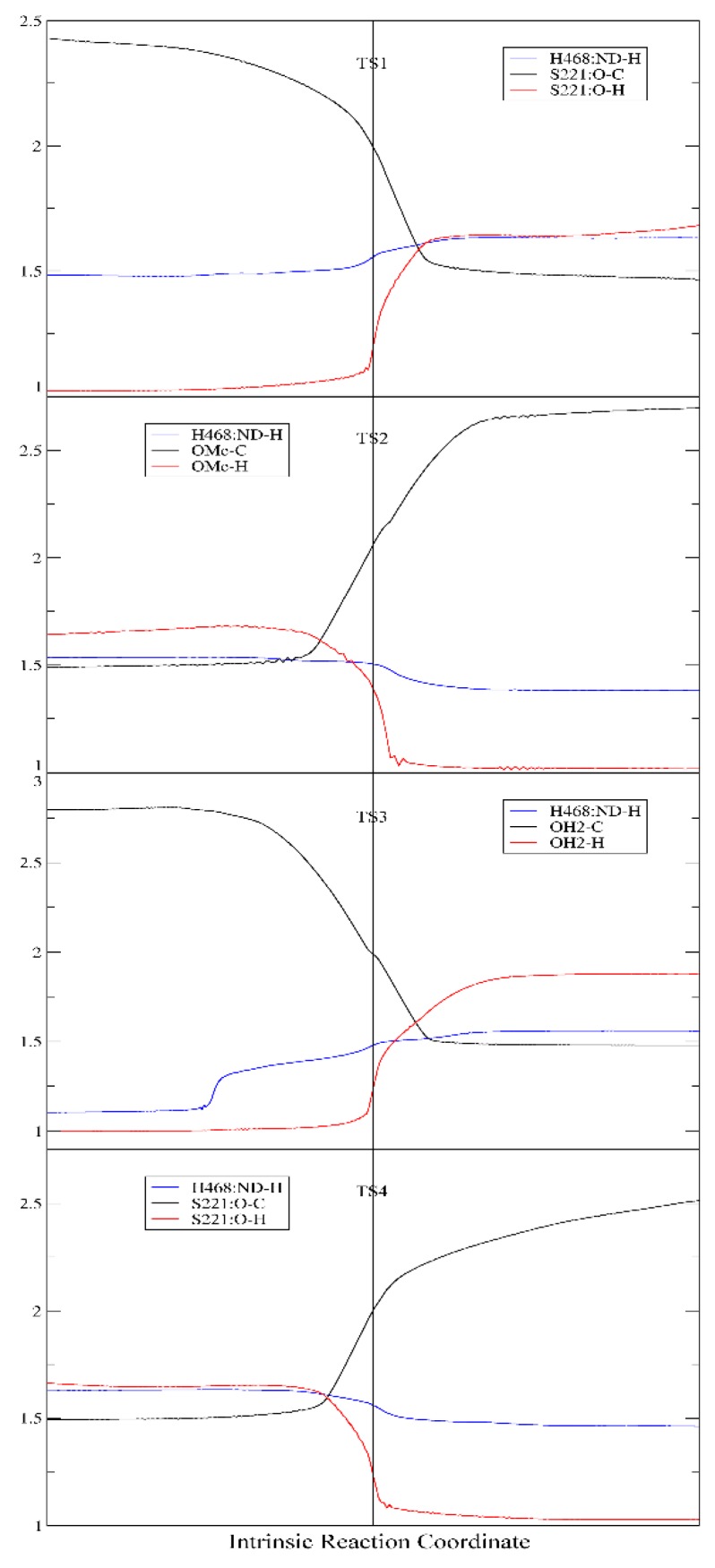
Distance (Å) of H468:Nδ–H (Blue), oxygen atom of S221/methanol/water–H (Red), and oxygen atom of S221/methanol/water–carbonyl carbon atom (Black) along the intrinsic reaction coordinate.

The second significant difference was that the proton transfer between H468 and E354 was observed in our study, but unobserved in the previous QM/MM study. Actually, in all carboxylesterases, the “catalytic triad” consisting of serine, glutamate, and histidine was highly conserved and played essential roles in the catalytic hydrolysis of the substrates, and mutation of any of these residues resulted in significant loss of the esterase activity [2,4,24,33]. According to the putative catalytic mechanism of carboxylesterases [4,24,25], the Glu and His residues in the catalytic triad formed a charge relay system, which efficiently assisted the proton transfer between the catalytic serine and alcohol/water molecule. Take the acylation stage as an example: in Step 1, the glutamate anion firstly deprived the proton from His:Nδ, then His:Nε deprived the proton from Ser:OH to produce a Ser–O anion, which then attacked the carbonyl carbon of the substrate to form the tetrahedron intermediate. Step 2 (releasing of the alcohol) was virtually a reversed process of Step 1; however, His:Nε would not return its proton (which was originally from Ser:OH) to serine, but transferred it to the alcohol, which gradually left the carbonyl carbon. The proposed proton transfer between Glu and His residues was clearly observed in this study. Nevertheless, our computation results show that the proposed three “substeps”, i.e., the H468→E354 proton transfer, the S221→H468 proton transfer, and the attacking of S221 to the carbonyl carbon, were roughly synchronous (Figure 6), instead of occurring sequentially. As shown in Figure 6, in all of the four steps, the three “substeps”, proton transfer between H468 and E354, proton transfer between H468 and S221/methanol/water, and attacking/leaving of oxygen of S221/methanol/water, occurred roughly in the same time period around the transition states. Obviously, upon receiving a proton from S221 (or water), H468 would carry a positive charge and thus become energetically less stable; so, by transferring another proton to E354 carboxylate anion, H468 would diminish its extra positive charge and reduce its energy. Contrary to our results, the previous QM/MM computation results [23] detected no proton transfers between H468 and E354, either in the acylation stage or in the deacylation stage. In the one-step acylation mechanism, H468 received a proton from S221 and immediately transferred it to the methoxy group, and the deacylation mechanism was similar to the acylation. Just from a chemical point of view, this proton transfer mechanism might also work; however, without such an H468 ↔ E354 proton transfer to alleviate the positive charge carried by H468, the tetrahedral “transition state” may be energetically unfavorable and the energy barrier of the reaction would possibly be increased.

### 2.5. Orthoester Intermediates in the hCES1-Catalyzed Hydrolysis of Cocaine

Our study found that orthoester intermediates existed in the hCES1-catalyzed cocaine hydrolysis process, both in the acylation stage (IM1) and in the deacylation stage (IM3). To our knowledge, a similar mechanism has been proposed for aminoacyl migration between 1,2-diol of nucleotides [34], but such orthoester intermediates have not been reported for enzymatic ester hydrolysis yet. The formation of orthoesters significantly reduced the energy of the intermediates, and hence elevated the free energy barriers of Steps 2 and 4 (Figure 1). As illustrated in Figure 1, if the reaction of Step 1 stopped at the tetrahedral intermediate IM1t without continuing to form orthoester intermediate IM1, the free energy barrier of Step 2 would be 16.7 kcal/mol, instead of 22.7 kcal/mol. Even so, Step 2 would still determine the rate of the whole cocaine hydrolysis reaction. This agrees with the previous DFT computation results of ester hydrolysis mechanism catalyzed by AeCXE1 [20], which showed that the second step (cleaving of the alkoxy group from the carbonyl) was the rate-determining step. Hence, without orthoester intermediates formed during the enzymatic reaction, the activation free energy (*E*_a_) of the whole reaction would be 16.7 kcal/mol at 298 K; according to the conventional transition state theory [35], the rate constant *k*_cat_ would be 3.6 s^−1^ (214 min^−1^), significantly higher than the actual *k*_cat_ value (0.058 min^−1^ [23]).

As we know, the hCES1-catalyzed hydrolysis of cocaine into methanol and benzoylecgonine (*k*_cat_ = 0.058 min^−1^ [23]) was significantly slower than the hydrolysis of cocaine into benzoic acid and ecgonine methyl ester (catalyzed by cocaine esterase or butyrylcholinesterase, *k*_cat_ = 7.8 s^−1^ and 4 min^−1^, respectively [36,37]). From a chemistry viewpoint, cleavage of the methyl ester bond of cocaine would be much easier than cleavage of the benzoate ester bond; this can also be supported by the fact that conversion of cocaine to benzoylecgonine could occur non-enzymatically in plasma, but conversion of cocaine to ecgonine methyl ester must be enzymatic [38]. Hence, we propose that the low reaction rate of hCES1-catalyzed cocaine hydrolysis might be partially attributed to the formation of orthoester intermediate during the enzymatic reaction.

**Figure 7:**
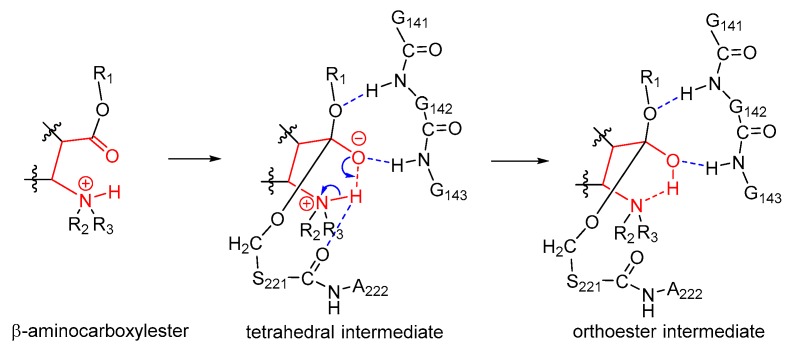
Proposed mechanism of formation of orthoester intermediates during the hCES1-catalyzed hydrolysis of substrates bearing β-aminocarboxylester structure. The intramolecular six-membered ring scaffold (shown in red) is chemically stable. Only the orthoester intermediate in the acylation stage is shown here for example.

Apparently, the unique chemical structure of the substrate cocaine was required during the formation of orthoester intermediates. Cocaine was actually a β-aminocarboxylester; as shown in Figure 2, upon formation of the tetrahedral intermediate IM1t, the negatively charged carbonyl oxygen atom attracted the hydrogen atom on the β-amino group by a hydrogen bond, and an intramolecular six-membered ring was formed. Such an intramolecular six-membered ring was rather stable and facilitated the proton transfer from the nitrogen atom to the oxygen atom to give the orthoester intermediate IM1. On the other hand, the unique steric/electrostatic restraints imposed by the catalytic cavity of hCES1 were important for keeping the appropriate substrate conformation required for the formation of the intramolecular six-membered ring, and hence were also important for the formation of the orthoester intermediate. Therefore, from this finding, we can further propose that other hCES1 substrates bearing the similar structure features (β-aminocarboxylesters) might also form orthoester intermediates in hCES1 catalytic cavity (Figure 7), and hence their hydrolysis rates might be decreased and their in vivo half-life prolonged. It should be emphasized that, since it is difficult to determine or characterize the detailed conformation of substrate in the covalent enzyme–substrate complex, the existence of orthoester intermediates of cocaine or other similar substrates still needs to be validated in future experimental studies. Nevertheless, we expect that this hypothesis can provide insights or clues for the study of metabolism mechanisms of other hCES1 substrates, as well as the design of drugs which need to be activated or deactivated by hCES1.

Furthermore, as stated above, polarization functions of hydrogen atoms were required to correctly describe the formation of orthoester intermediates. In most QM cluster or QM/MM computation studies of enzymatic reactions, polarization functions of hydrogen atoms were usually omitted and this did not seem to cause great problems. But based on this finding, we would recommend that polarization functions should be applied to hydrogen atoms, at least those directly involved in the proton transfer, to correctly describe the reaction mechanisms and to explore more details of the reaction.

## 3. Materials and Methods

### 3.1. Construction of Cluster Model of hCES1–Cocaine Complex

The X-ray crystal structure of hCES1 (PDB entry ID: 1MX5 [17]) was used as the initial three-dimensional (3-D) structure for the modeling, because its ligand homatropine has the same scaffold (tropine) to cocaine and just differs from cocaine in the sidechains. The structure of homatropine was then converted to cocaine by modifying the sidechains (including adding the methoxycarbonyl group and removing the hydroxymethylene group). The nitrogen atom of cocaine was protonated and the amino acid residues were set to usual protonation states. Sidechains of a few amino acid residues were slightly adjusted and an energy-minimization was performed to relax the system. Cocaine and the surrounding amino acid residues, namely Leu97, Gly141–Gly143, Ser221–Ala222, Leu255, Glu354, Leu363, Phe426, and His468, were selected to be included in the final cluster model for QM computations (176 atoms in total). The final QM cluster model had a net charge of 0 and a multiplicity of 1. The whole preparation process of the cluster model was accomplished in BIOVIA Discovery Studio 2017 [39].

### 3.2. Computation of the Reaction Mechanism and Free Energies

The reaction mechanism and the free energy profiles of the enzymatic reaction were calculated using conventional quantum chemistry methods. For each elementary reaction, the transition state (TS) was firstly obtained by geometrical optimization of the initial guess structure, and the intrinsic reaction coordinate (IRC) was calculated, followed by geometrical optimization to give the reactant, product, and intermediates. To avoid the distortion of the model, the carbon α (C_α_) atom of each amino acid residue was frozen during the simulation. All the stationary point structures were then subject to vibration analysis and calculated for the thermal correction values at both 298 K and 310 K. The hybrid-meta GGA functional M06-2X [40,41] was used in the geometrical optimization, vibration analysis, and IRC calculation, and DFT-D3 empirical dispersion corrections (zero damping) [42] were applied in the calculation. Cocaine and the residues participated in the catalytic reaction or closed to the reaction center (Glu354, His468, Ser221, Ala222, and Gly141–Gly143) were assigned the basis set 6–31G* [43,44,45], and other residues which were non-polar and far from the reaction center (Leu97, Leu255, Leu363, and Phe426) were assigned the basis set 3–21G [46]. Hydrogen atoms involved in the proton transfer or hydrogen bonding were assigned the basis set 6–31G** [43,44,45]. The frequency scale factor of 0.9670 [47] was used in the calculation of thermal correction values. All the above QM computations were done in Gaussian 16 [48] using the IEFPCM implicit solvent model [49,50,51]. Finally, single point energy of all the stationary point structures were recalculated using the double-hybrid functional PWPB95 [52] and basis set def2-TZVP [53,54] with DFT-D3 corrections (BJ damping) [55], which were added to the thermal correction values of Gibbs free energy to give the final free energies. This step of single point energy calculation was accomplished in ORCA 4.1.1 [56,57] with density-fitting approximation [54,58,59] and the CPCM solvent model [60]. The dielectric constant (ε) of the solvent in both Gaussian 16 and ORCA 4.1.1 was set to 4 to mimic the protein environment around the catalytic site [20,27,61,62].

## 4. Conclusions

In this study, the reaction mechanism of hCES1-catalyzed hydrolysis of cocaine was investigated by the QM cluster method. Our results clearly show a four-step hydrolysis mechanism, in which both the acylation stage and the deacylation stage include two transition states and one tetrahedral orthoester intermediate. Moreover, this study found that orthoester intermediates exist in the hCES1-catalyzed hydrolysis process of cocaine, which significantly elevate the activation free energy of the reaction and decrease the reaction rate. We further propose that hCES1 substrates with β-aminocarboxylester structure might undergo a similar orthoester mechanism, and hence their metabolism would be delayed. We expect that this finding and hypothesis may provide clues or ideas for the study of hCES1-related drug metabolism and design of drugs which need to be metabolized or activated by hCES1.

## Supplementary Materials

The structures of Reactant, transition states, intermediates, and product can be found in the Electronic Supplementary Information.
