# Supplementary material for: Catalytic Hydrolysis Mechanism of Cocaine by Human Carboxylesterase 1: An Orthoester Intermediate Slows Down the Reaction
Source: Molecules. 2019 Nov 9;24(22):4057. doi: 10.3390/molecules24224057 (PMC6891567; doi:10.3390/molecules24224057)
Supplement: Supplementary file 1 [file molecules-24-04057-s001.pdf]

Catalytic hydrolysis mechanism of cocaine by human carboxylesterase 1: an orthoester intermediate slows down the reaction

Maocai Yan [1,2,*], Zhen Zhang [1], Zhaoming Liu [1], Chunyan Zhang [1], Jingchang Zhang [1], Shuai Fan [2,3] and Zhaoyong Yang [3,*]

[1] School of Pharmacy, Jining Medical University, Rizhao 276800, China
[2] Beijing Key Laboratory of Antimicrobial Agents, Institute of Medicinal Biotechnology, Chinese Academy of Medical Sciences and Peking Union Medical College, Beijing 100050, China
[3] Institute of Medicinal Biotechnology, Chinese Academy of Medical Sciences and Peking Union Medical College, Beijing 100050, China
* Correspondence: maocaiyan@mail.jnmc.edu.cn (M.Y.); zhaoyongy@imb.pumc.edu.cn (Z.Y.); Tel.: +86-(633)-2983781 (M.Y.), +86-(10)-63165283 (Z.Y.)

Supplementary Information

Figure S1 ~ S12. Structures of Reactant, transition states, intermediates, and Product. The bold green dash lines indicate hydrogen bonds. The bond lengths and atom distances are given in angstrom (Å). The amino acid residues which are not directly involved in the catalytic reaction are displayed in line model.

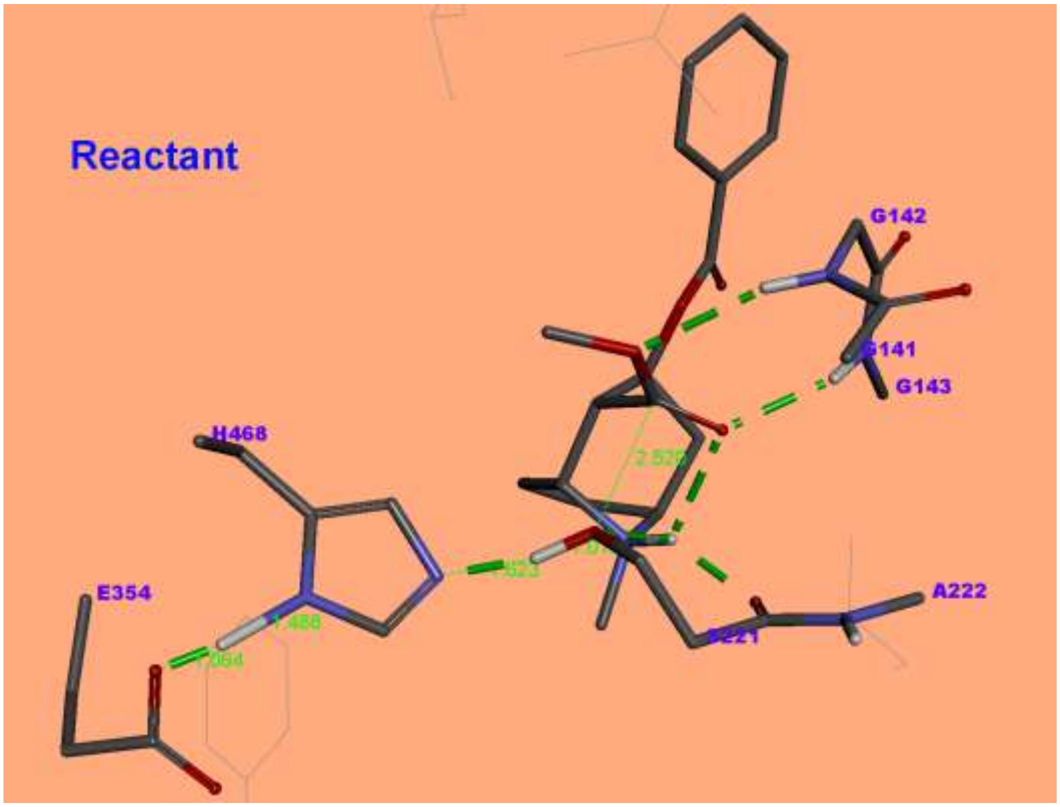

Figure S1. Structure of the Reactant.

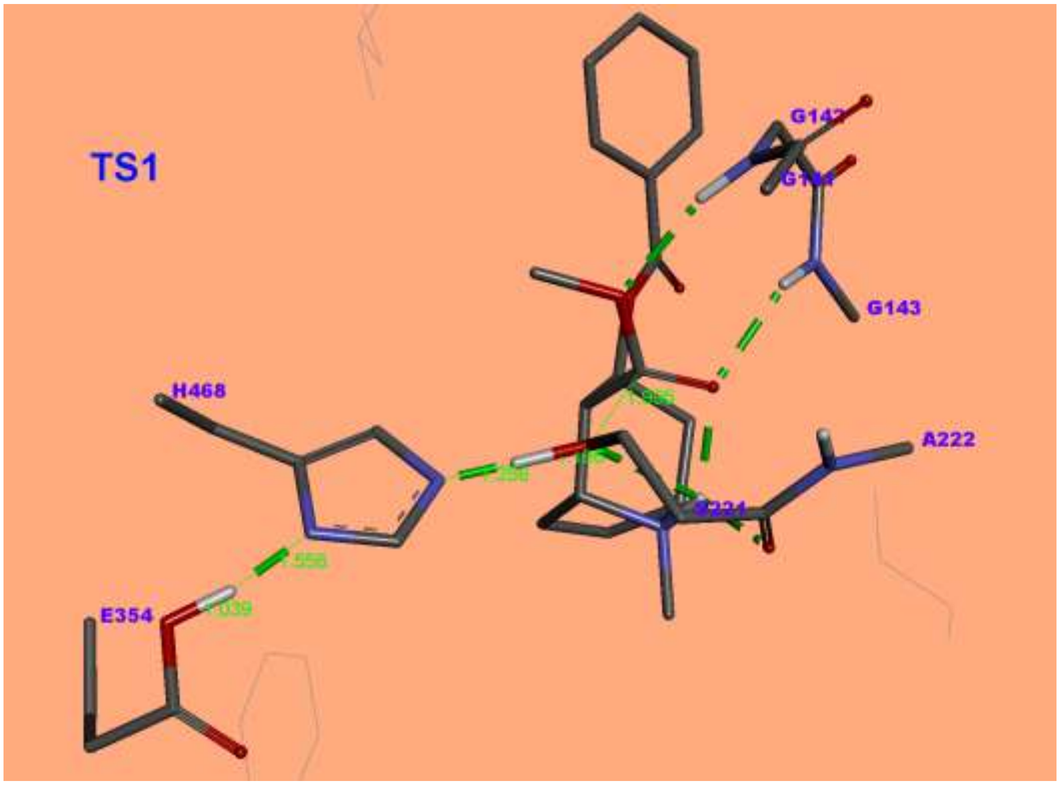

Figure S2. Structure of the transition state TS1.

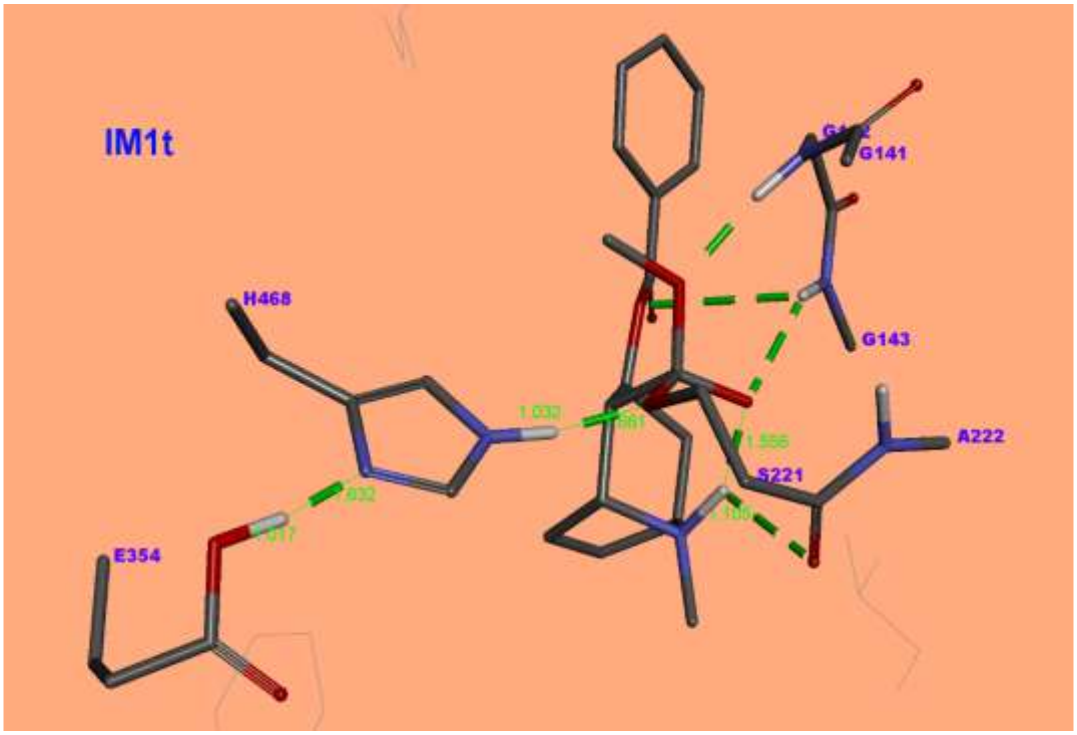

Figure S3. Structure of tetrahedral intermediate IM1t.

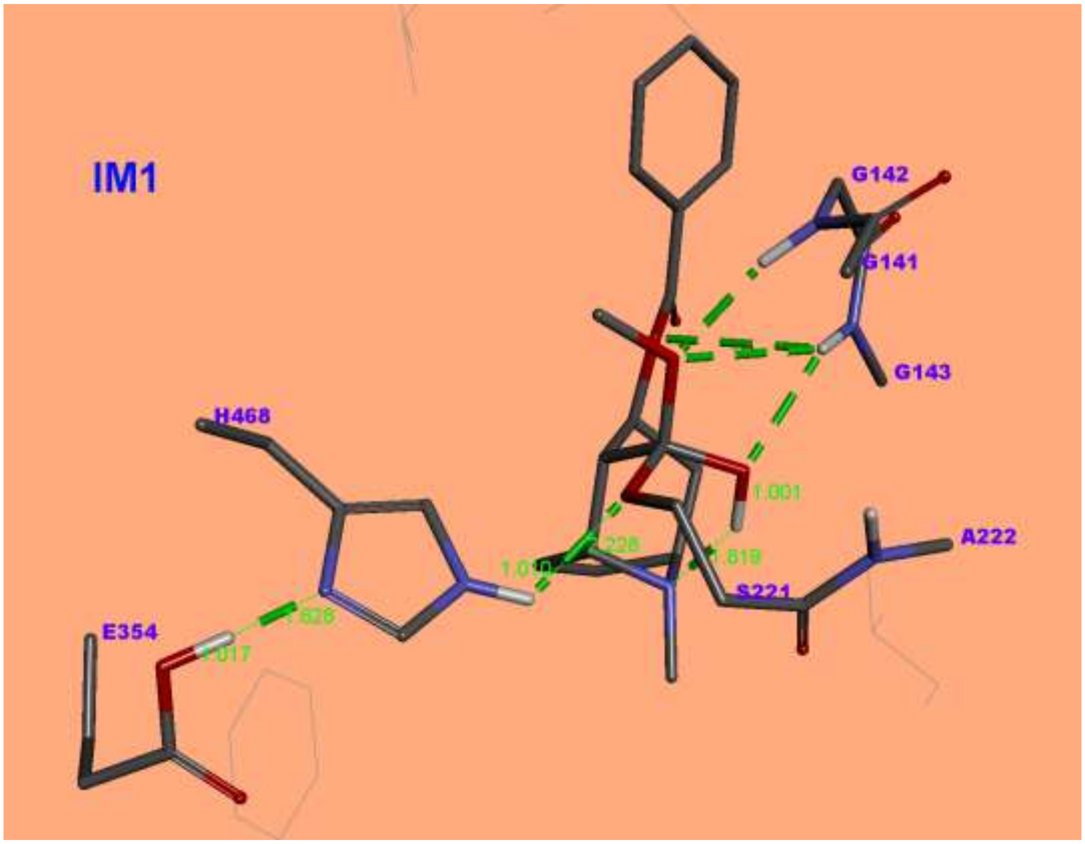

Figure S4. Structure of orthoester intermediate IM1.

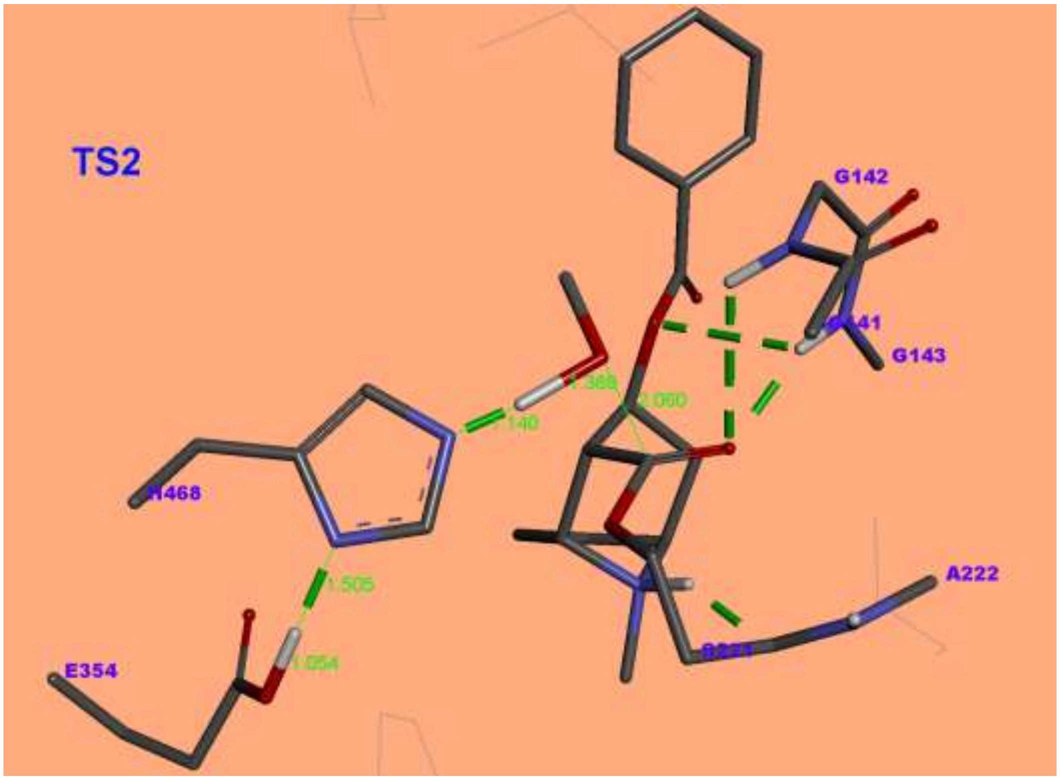

Figure S5. Structure of transition state TS2.

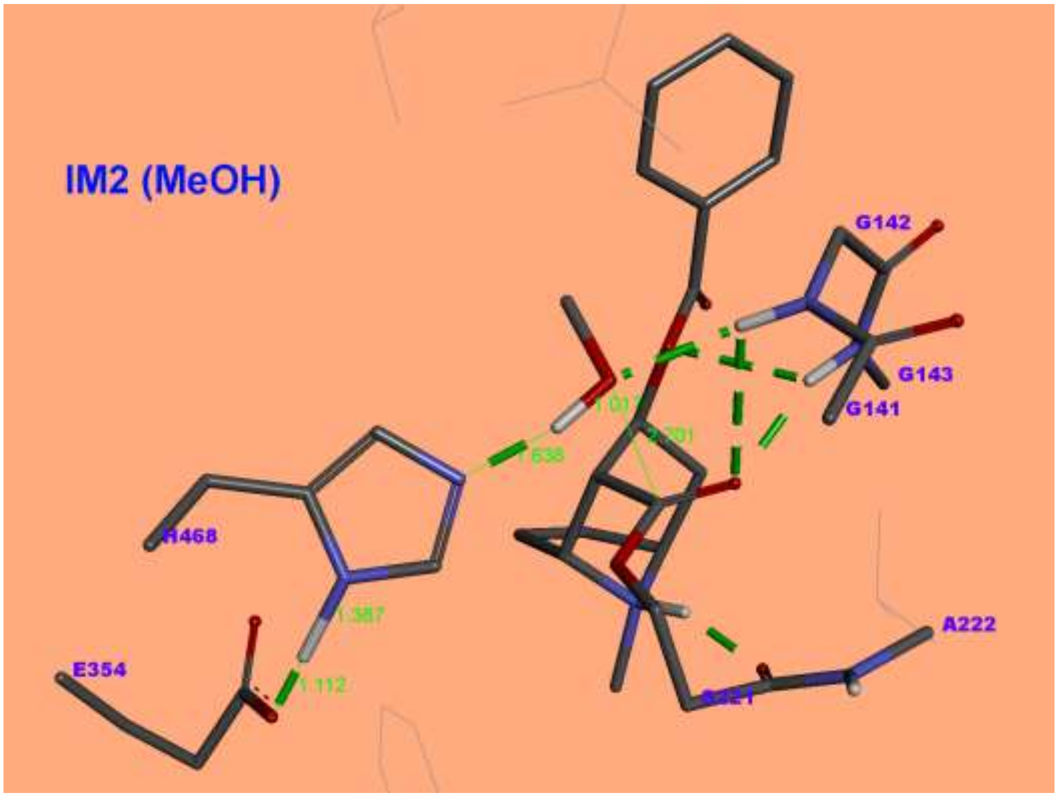

Figure S6. Structure of intermediate IM2 (acyl-hCES1 with methanol).

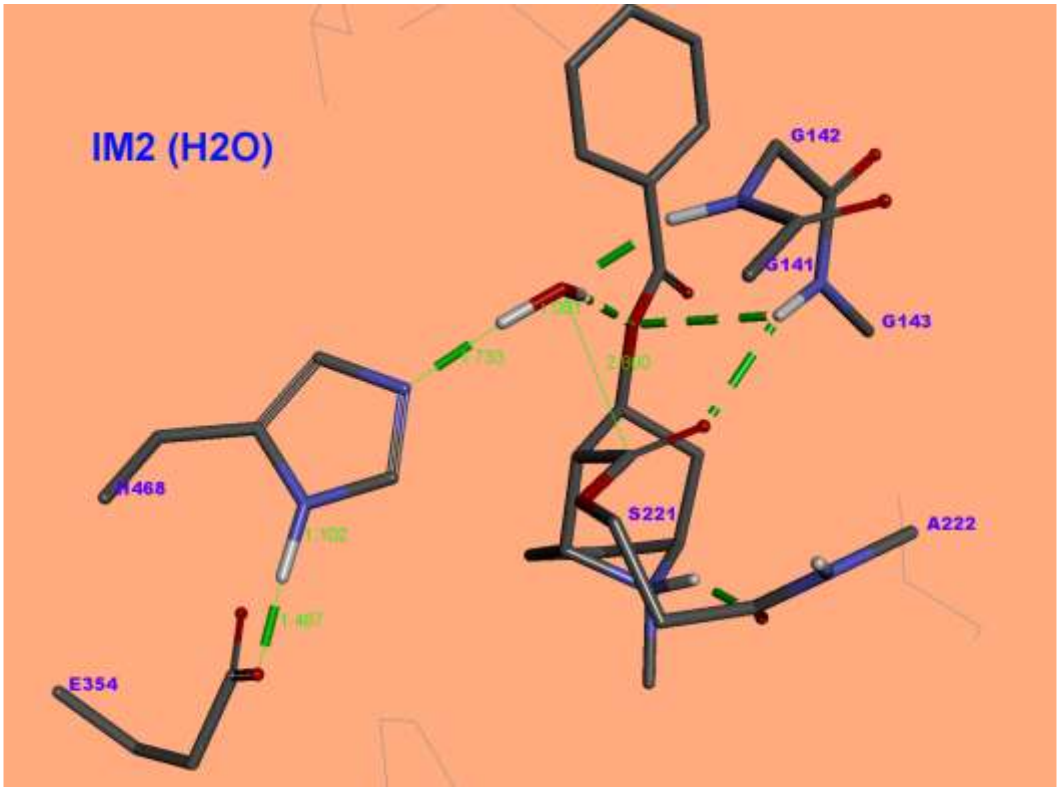

Figure S7. Structure of intermediate IM2 (acyl-hCES1 with water).

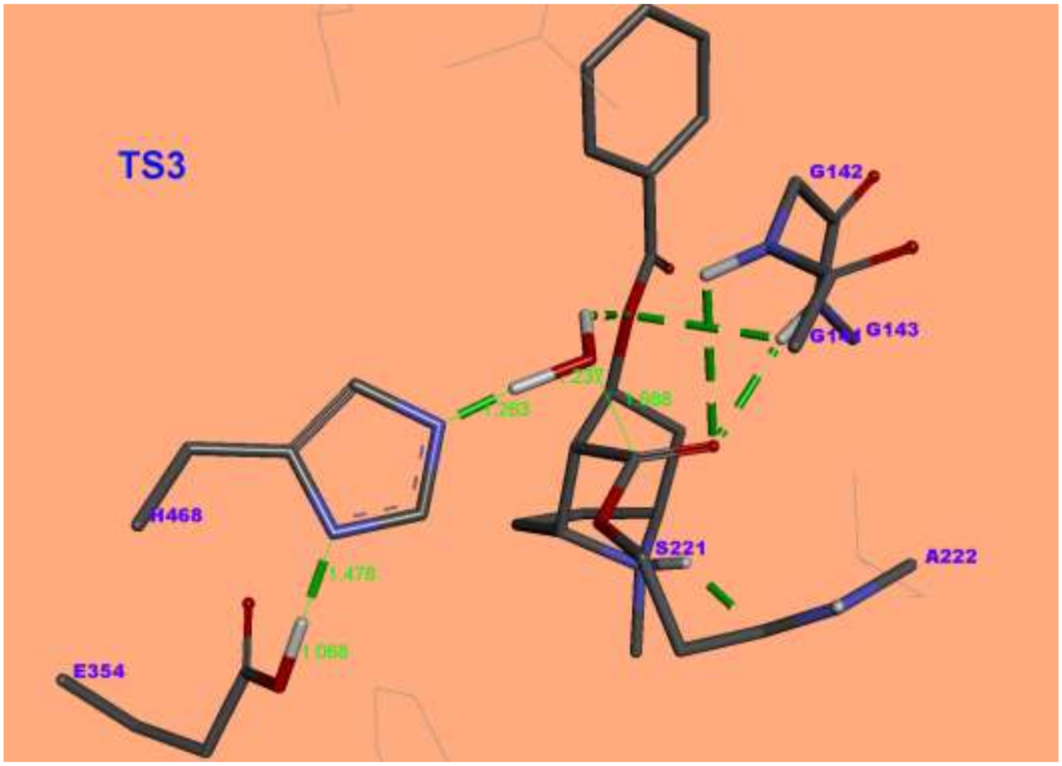

Figure S8. Structure of transition state TS3.

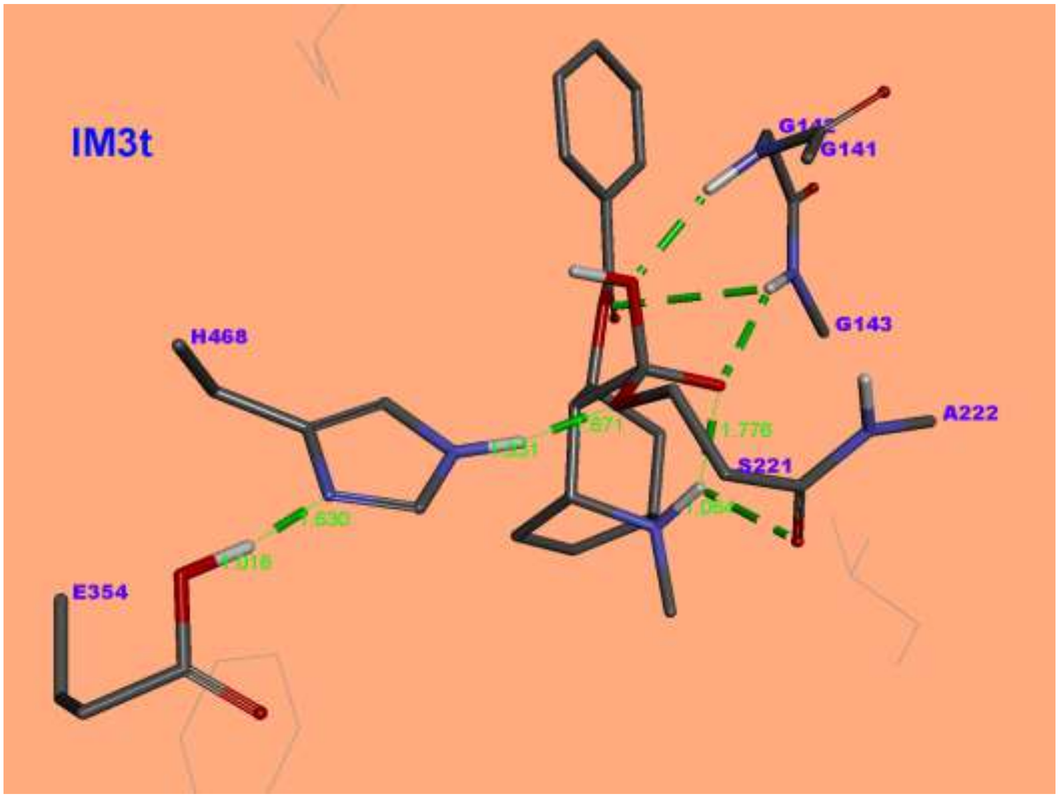

Figure S9. Structure of tetrahedral intermediate IM3t.

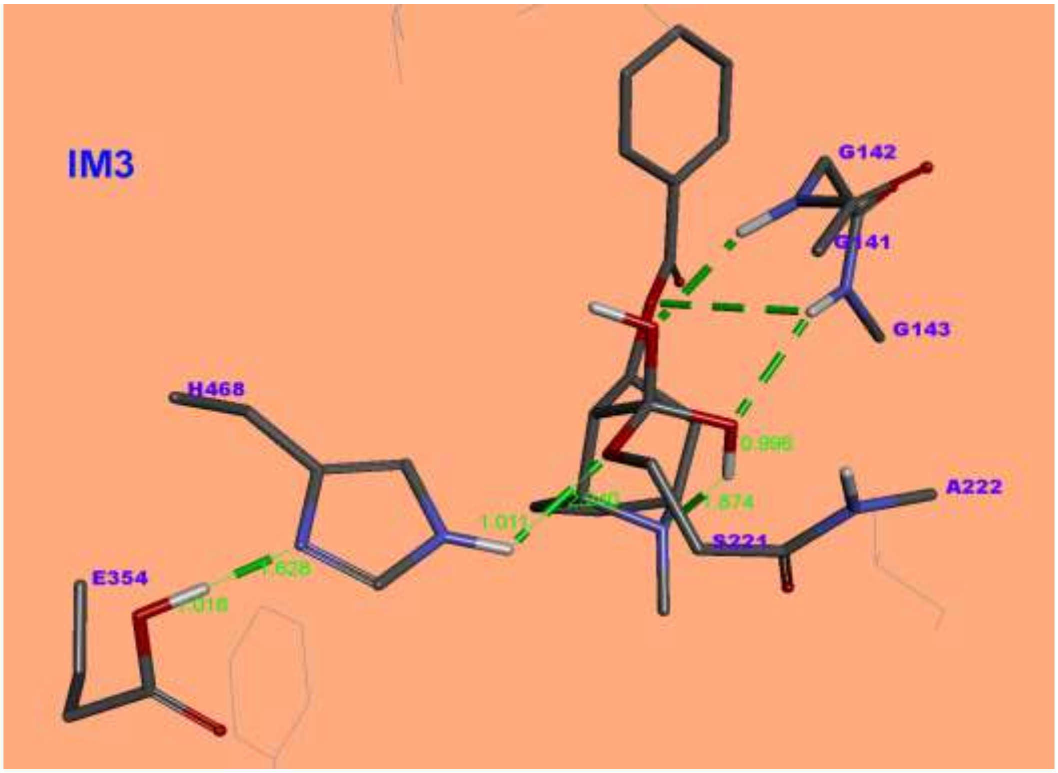

Figure S10. Structure of orthoester intermediate IM3.

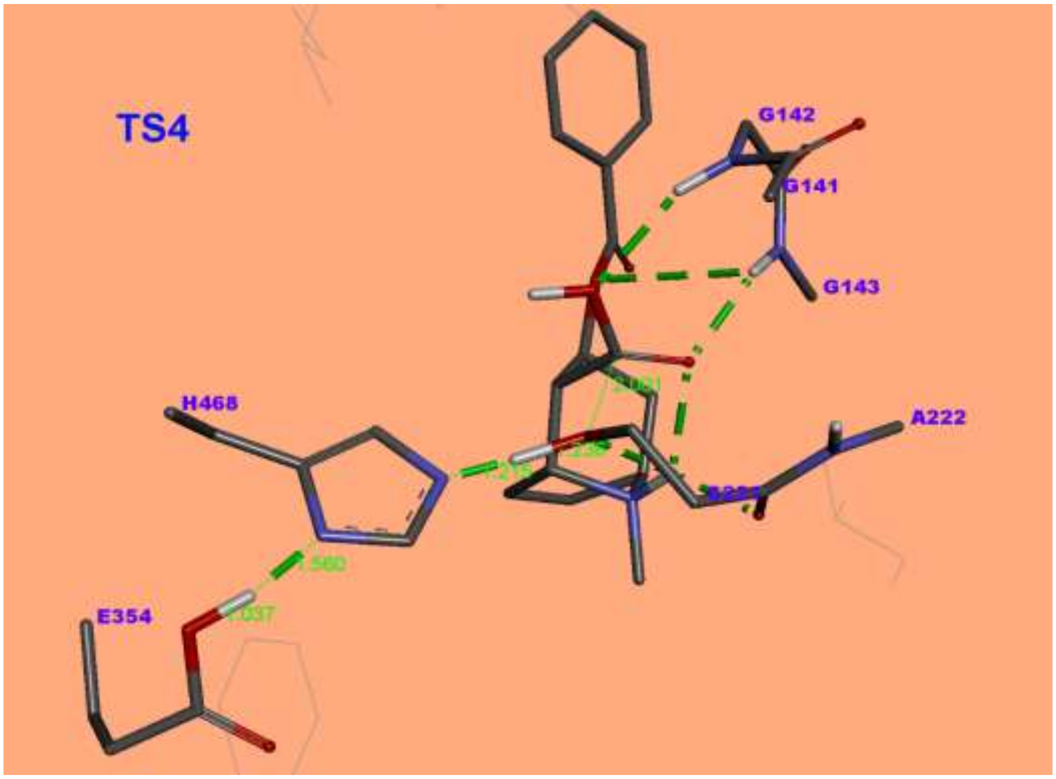

Figure S11. Structure of transition state TS4.

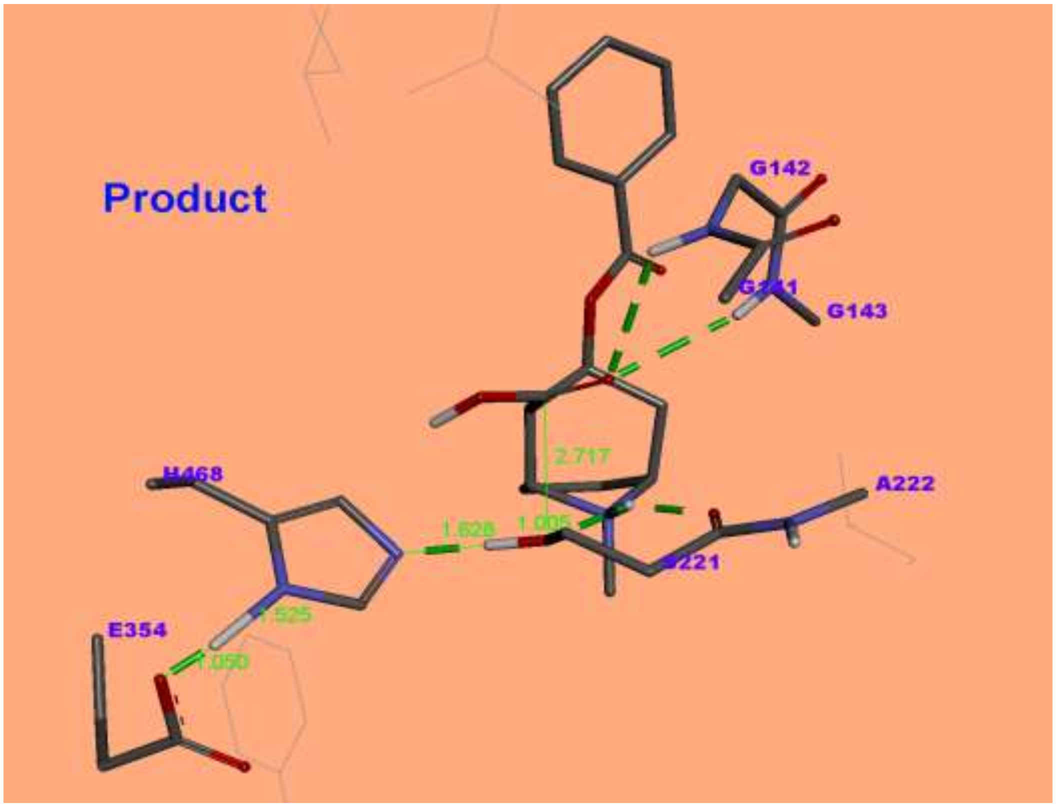

Figure S12. Structure of the Product.
